# Phosphaturic Mesenchymal Tumor: A Case Report of a Rare Entity

**DOI:** 10.7759/cureus.22712

**Published:** 2022-02-28

**Authors:** Rawan E Hudairy, Abdelrazak Meliti, Ghadeer Mokhtar, Maram Alanazi

**Affiliations:** 1 Anatomic Pathology, King Faisal Specialist Hospital and Research Centre, Jeddah, SAU

**Keywords:** hypophosphatemia, grungy basophilic matrix, paraneoplastic syndrome, tumor-induced osteomalacia, phosphaturic mesenchymal tumor

## Abstract

Phosphaturic mesenchymal tumor (PMT) is a rare entity that presents as a paraneoplastic syndrome that causes tumor-induced osteomalacia (TIO). Most of these cases were located in the extremities. We report a case of a phosphaturic mesenchymal tumor arising in the left upper thigh. The tumor was discovered during the clinical workup of a patient complaining of osteomalacia symptoms with multiple fractures.

## Introduction

Phosphaturic mesenchymal tumor (PMT), also known as tumor-induced osteomalacia (TIO), is a rare paraneoplastic syndrome caused by mesenchymal tumors secreting fibroblast growth factor-23 (FGF-23) [1-3]. In 1947, TIO was first reported by McCance in a 15-year-old girl who presented with symptoms of weakness and gait disturbance along with hypophosphatemia. PMT was included in the 2013 WHO classification of tumors of the soft tissue and bone [4]. The term PMT was first coined by Weidner and Santa Cruz in 1987 [5]. Up to date, fewer than 500 cases have been reported. Most PMT cases were located in the extremities. Herein, we report an additional case of PMT-induced osteomalacia in a 48-year-old woman.

## Case presentation

A 48-year-old female who was a known case of diabetes mellitus presented to our hospital with right hip pain, inability to bear weight, and a history of multiple unprovoked fractures. Laboratory workup showed hypophosphatemia (0.26 mmol/L), low 1,25-hydroxy vitamin D (65 nmol/L), high FGF-23 (1,029 kRU/L), high alkaline phosphatase level (525.0 U/L), normal serum calcium level (2.24 mmol/L), and high 24-h urine phosphate (53 mmol/day) (Table 1).

**Table 1:** Laboratory workup results.

Parameters/variables			Patient test result			Normal range		
PO4			0.26 mmol/L			0.97–1.45 mmol/L		
Vitamin D25 hydroxy, total			65 nmol/L			75–250 nmol/L		
FGF-23c-terminal			1092 kRU/L			26–110 kRU/L		
Alkaline phosphatase			525 U/L			46–122 U/L		
Calcium			2.24 mmol/L			2.10–2.55 mmol/L		
PO_4_, 24 hr Urine			53 mmol/day			13–42 mmol/day		

**Figure 1:**
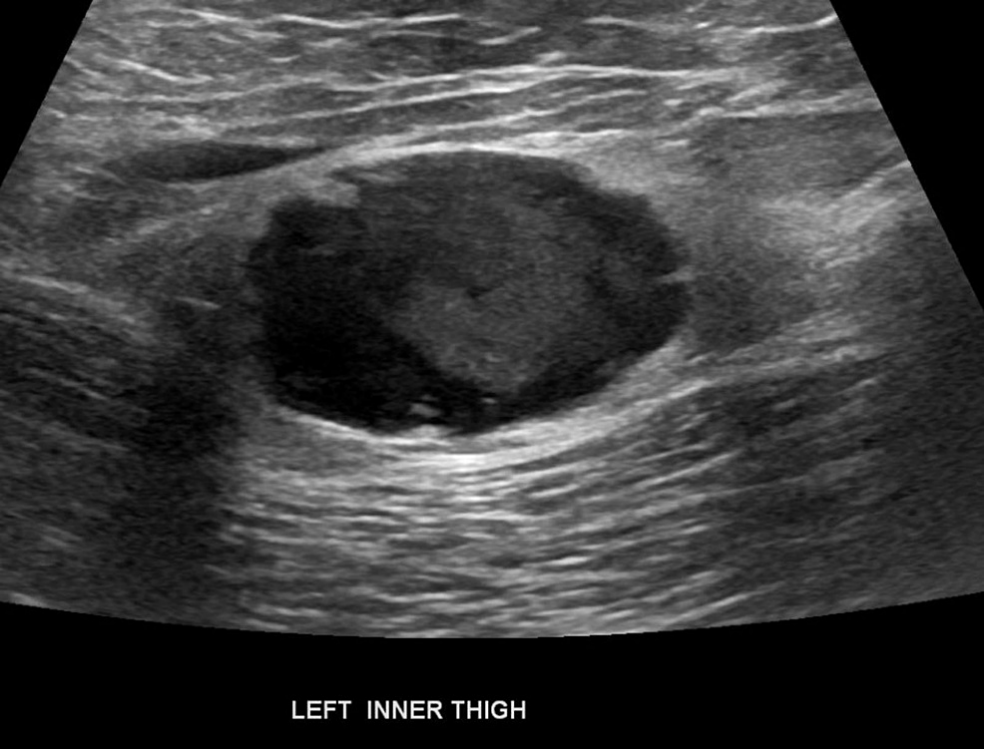
Ultrasonography showing hypoechoic mass.

Bone mass density (BMD) showed osteopenia with multiple fractures. The patient was evaluated by ultrasonography and was found to have a 3.2 × 2 cm^2^ heterogenous hypoechoic mass involving the medial aspect of the left thigh with increased vascularity (Figure 1).

**Figure 2:**
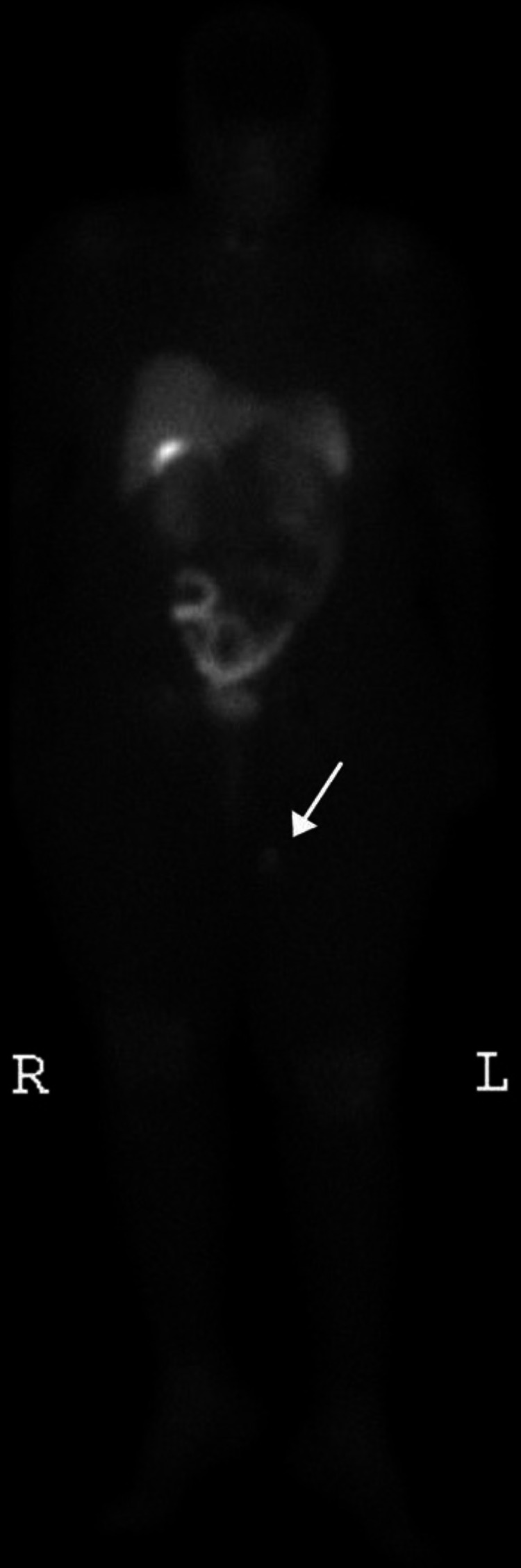
An Octreotide scan showed a hot lesion in the left upper thigh (white arrow).

No other bone or soft tissue masses were identified. An Octreotide scan showed a hot lesion in the left upper thigh (Figure 2).

**Figure 3:**
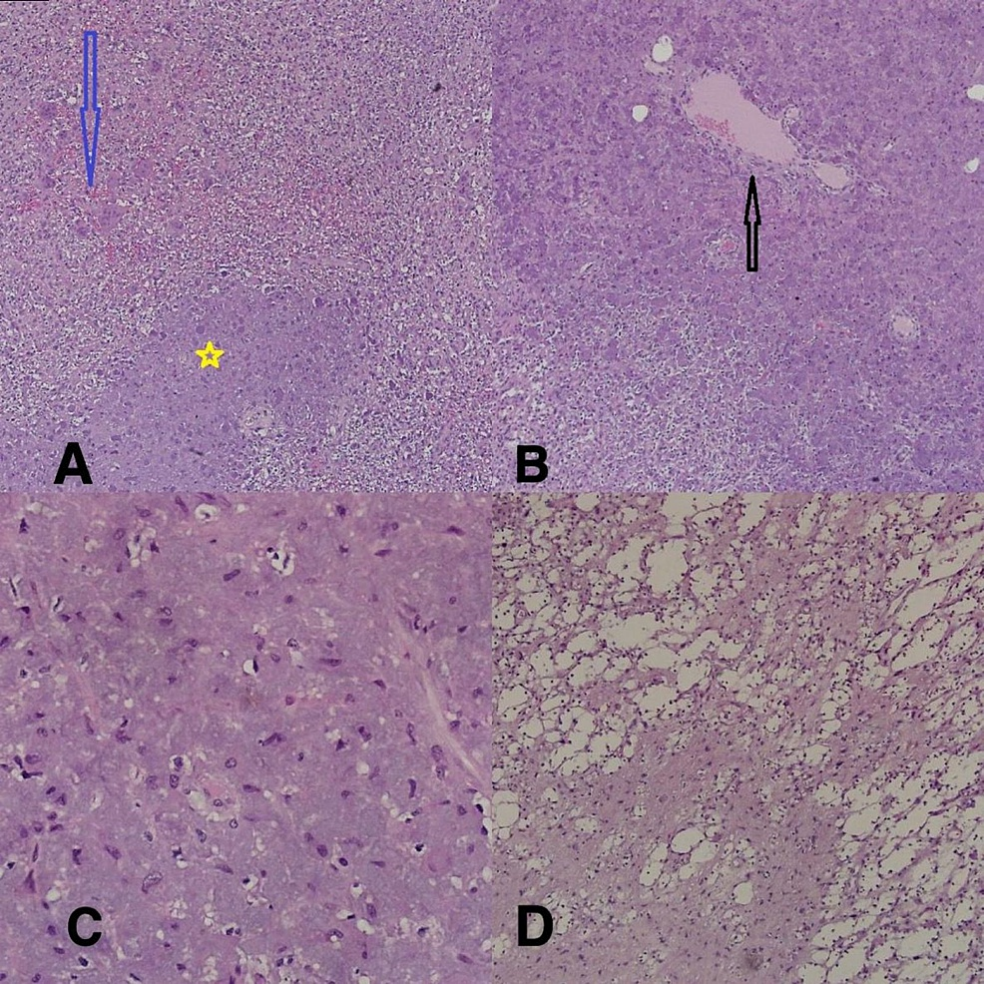
(A) and (B) H&E (4× and 10×) highlight the grungy smudgy basophilic calcifications (yellow asterisk), multinucleated giant cells (blue arrow), hemangiopericytomatous-like blood vessels (black arrow). (C) H&E (40×) demonstrates a bland proliferation of spindle cells, no evidence of hypercellularity, pleomorphism, or mitoses. (D) H&E (10×) highlights the microcystic changes.

During the evaluation, the patient was treated empirically with phosphate, calcium, and vitamin D supplements. The lesion on the left upper thigh was surgically excised. Histopathological examination revealed a bland spindle cell proliferation in a background of hemangiopericytomatous-like blood vessels, multinucleated giant cells, smudgy/grungy basophilic matrix, and microcystic changes with rare mitosis (1-2/10 HPF). There was no evidence of pleomorphism, hyper-cellularity, or necrosis (Figure 3A-3D).

**Figure 4:**
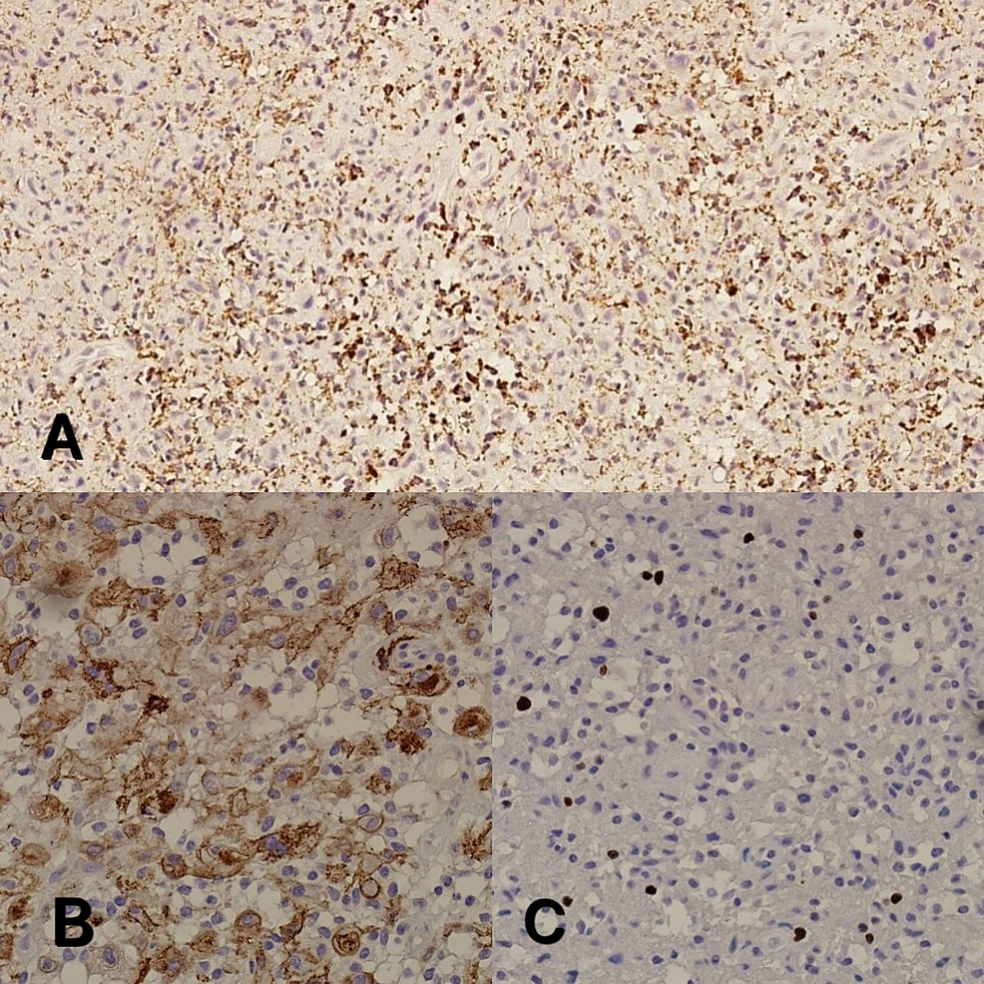
Immunohistochemistry; (A) CD68 (10×), (B) CD56 (20×), (C) Ki-67 shows very low proliferative index (20×).

A panel of immunohistochemistry was performed, including vimentin, CD68, CD56, CD34, ERG, and Ki67. The tumor cells showed positive immunoreactivity against ERG and CD56, with variables for CD68, while vimentin and CD34 were negative. Ki67 showed a very low proliferative index (<5%) (Figure 4A-4C). In light of the clinical data and based on the above morphological features and the immunoprofile, the patient was diagnosed with phosphaturic mesenchymal tumor-induced osteomalacia.

## Discussion

Phosphaturic mesenchymal tumors are an infrequent entity that is usually present as a paraneoplastic syndrome that causes tumor-induced osteomalacia (TIO) [1-3]. Osteomalacia is a metabolic disorder characterized by insufficient mineralization of mature bone, most commonly due to vitamin D deficiency and less frequently due to inborn errors and chronic kidney disease. Other much less frequent causes of tumor-induced osteomalacia include osteoblastoma, osteosarcoma, hemangiopericytoma, plasmacytoma, fibroma, hemangioma, and giant cells. The pathogenesis of osteomalacia is secondary to the production of phosphate-regulating substances by the tumor cells, most importantly the FGF-23. FGF-23 diminishes the ability of proximal renal tubules to reabsorb phosphate and increases renal excretion of the phosphate. In addition, it increases bone resorption of both calcium and phosphate, decreases intestinal absorption of calcium and phosphate, and inhibits 1-α-hydroxylase production of 1,25 dihydroxycholecalciferol as well. Therefore, it causes decreased bone mineralization. Other regulating proteins secreted by PMT tumor cells are FGF-7, secreted frizzled-related protein 4 (sFRP-4), dentin matrix protein 1 (DMP1), and matrix extracellular phosphor-glycoprotein (MEPE) [4-7].

Phosphaturic mesenchymal tumors are found to be the most common cause of TIO [8]. PMT characteristically affects the extremities of middle-aged patients (30 to 40 years old) with no gender predilection. The tumor size is usually less than 5 cm, with 40-45% of the tumors originating from bone and 50-55% arising from soft tissues. Up to date, fewer than 500 cases have been reported. PMT diagnosis is usually delayed, attributed to its vague clinical symptoms, relatively slow growth, and small-sized lesions, which usually contribute to the difficulty in tumor localization [9]. Secondary TIO has been reported with other tumors such as small cell carcinoma, neurofibromatosis, colon adenocarcinoma, and ovarian cancer. The clinical presentation includes osteomalacia, joint and muscle aches, generalized weakness, and pathologic fractures [10]. In the pediatric age group, rickets and growth retardation are the manifestations of the disease.

The laboratory findings observed in PMT-induced osteomalacia are hypophosphatemia and relative hyperphosphaturia, the most consistent biochemical derangements. In addition, increased levels of intact FGF-23, low-normal serum calcium and parathormone levels, and a decrease in 1,25 dihydroxyvitamin D are also noted. However, a non-phosphaturic state has also been described [11]. Therefore, the absence of osteomalacia does not exclude the diagnosis of PMT. There is an elevated level of serum alkaline phosphatase as a result of increased bone turnover.

Different imaging modalities are used to localize the tumor. Whole-body Tc-99m sestamibi scanning, octreotide scintigraphy, 18F-fluorodeoxyglucose (FDG) positron emission tomography (PET), whole-body MRI, and computed tomography (CT) are used for the localization of these tumors. It is difficult to recommend one modality over another, and the choice primarily depends on availability [12]. CT typically shows a non-specific pattern of a hypodense round/circular mass with well-identified borders.

Microscopically, four variants of PMTs exist: osteoblastoma-like variant, non-ossifying fibroma-like variant, ossifying fibroma-like variant, and mixed connective tissue variant [1,4]. The most common variant is the mixed connective tissue type. The morphological differential diagnosis includes other mesenchymal tumors such as chondromyxoid fibroma, chondroblastoma, aneurysmal bone cyst, and osteosarcoma.

PMTs' histological features are characterized by the presence of grungy, smudgy basophilic calcifications, bland-looking normochromic spindle cells with small nuclei, and inconspicuous nucleoli in a myxoid background and very few mitotic figures. Additional morphological features such as osteoid matrix, multinucleated giant cells, hemangiopericytoma-like blood vessels, microcystic changes, and entrapment of mature adipose tissue can be seen. None of the above histological features are pathognomonic in and of themselves, but in the right clinical context and with the laboratory findings, they support PMT diagnosis [1,3-5]. The immunoprofile demonstrates positive reactivity for vimentin and FGF-23 with 100% specificity. Smooth muscle actin and somatostatin receptors are positive as well [4]. Other markers such as desmin, leu-M1, cytokeratin, leukocyte common antigen, and factor VIII-related antigen are usually negative in tumor cells. FGF-23 expression can also be detected by the reverse transcription-polymerase chain reaction (RT-PCR) technique. However, some tumors, such as fibrous dysplasia, chondromyxoid fibroma, and aneurysmal bone cysts, are found to produce a minimum amount of FGF-23 and may result in a false-positive result. Although the ultrastructural features of PMT tumor cells are similar to those of neuroendocrine tumors, they usually lack the expression of neuroendocrine markers (S-100, neuron-specific enolase, chromogranin, and synaptophysin). Beygi et al. recognized that approximately 42% of PMT harbor a fibronectin 1-fibroblast growth factor receptor 1 (FN1-FGFR1) fusion identified [4]. Additionally, a fusion of fibronectin and fibroblast growth factor receptor 1 (FN/FGF-1) has been reported in 6% of PMT cases, implying a targeted therapy approach in the future [2,4]. Complete surgical resection is the mainstay of treatment with a nearly 90% curative rate, with the achievement of negative surgical margins (10 mm) to reduce the chance of local recurrence. Resolution of symptoms (three to six months) with normalization of the biochemical FGF-23 serum level and other laboratory tests is usually achieved within a week following complete resection. Reversal of serum phosphate level and other biochemical markers following surgical resection is required to confirm the diagnosis of PMT with TIO [13].

Radiation therapy is used for tumors that are not amenable to surgical resection or incompletely resected tumors with positive margins. Radiofrequency ablation for small bony tumors in critical locations is advised. Medical treatment including phosphorus and calcitriol supplementation is proposed for cases where the tumor cannot be clinically localized. However, hyperparathyroidism, hypercalcemia, and nephrolithiasis could be potential complications of such medical treatment [3,4].

PMT is characteristically a benign tumor [4,5]. Local recurrence and malignant behavior have been reported in 15 patients. Metastasis, most commonly to the lung, has occasionally been reported [5,8]. Therefore, postoperative screening and long-term follow-up through serum levels of phosphate and FGF-23 as they correlate with recurrence is warranted [3,4,9,10]. No anticipating factors have been reported in the literature to predict malignant transformation at the early stages of PMT. The only clue for malignant transformation is the change in the tumor size or the number of tumor masses with a progressive increase in FGF-23 level and multiple metastases. Nevertheless, multiplicity has been reported in a benign PMT [5]. Variable histopathological appearances are described in malignant transformation, ranging from benign-looking to sarcomatoid features, but lacking the classic, typical PMT histology [4,5]. Thus, detection of FGF-23 mRNA in the tumor cells with increased FGF-23 levels is required to diagnose malignant PMT. A diffuse and robust positive immunoreactivity for p53 and the presence of a TP53 mutation have been identified in malignant PMT, suggesting that the TP53 mutation has a role in the malignant transformation of PMT. Similar to the benign counterpart, the malignant PMT is effectively treated with complete surgical resection. However, they are chemo-radio resistant.

## Conclusions

Phosphaturic mesenchymal tumor is an extremely rare neoplasm. PMT is considered one of the most common causes of tumor-induced osteomalacia (TIO) and is characteristically benign. We report an additional case of PMT-induced osteomalacia in a 48-year-old woman. The presence of hypophosphatemia, hyperphosphaturia, and a high level of fibroblast growth factor-23 with clinical symptoms of osteomalacia are essential clues for PMT diagnosis. The patient did well after surgery and was discharged in stable condition. A follow-up was done after six months, and there was no recurrence.

## References

[1] Richardson AL, Richardson OK (2019). Phosphaturic mesenchymal tumor: case report. Radiol Case Rep.

[2] Adnan Z, Nikomarov D, Weiler-Sagie M, Roguin Maor N (2019). Phosphaturic mesenchymal tumors among elderly patients: a case report and review of literature. Endocrinol Diabetes Metab Case Rep.

[3] Agarwal N, Kale SS, Kumari K (2019). Tumor-induced osteomalacia due to a phosphaturic mesenchymal tumor in the cervical spine: a case report and literature review. Neurol India.

[4] Beygi S, Denio A, Sharma TS (2017). The foot that broke both hips: a case report and literature review of tumor-induced osteomalacia. Case Rep Rheumatol.

[5] Oyama N, Kojima-Ishii K, Toda N (2020). Malignant transformation of phosphaturic mesenchymal tumor: a case report and literature review. Clin Pediatr Endocrinol.

[6] Hautmann AH, Hautmann MG, Kölbl O, Herr W, Fleck M (2015). Tumor-induced osteomalacia: an up-to-date review. Curr Rheumatol Rep.

[7] Ghorbani-Aghbolaghi A, Darrow MA, Wang T (2017). Phosphaturic mesenchymal tumor (PMT): exceptionally rare disease, yet crucial not to miss. Autops Case Rep.

[8] Folpe AL, Fanburg-Smith JC, Billings SD (2004). Most osteomalacia-associated mesenchymal tumors are a single histopathologic entity: an analysis of 32 cases and a comprehensive review of the literature. Am J Surg Pathol.

[9] Minisola S, Peacock M, Fukumoto S, Cipriani C, Pepe J, Tella SH, Collins MT (2017). Tumour-induced osteomalacia. Nat Rev Dis Primers.

[10] Ledford CK, Zelenski NA, Cardona DM, Brigman BE, Eward WC (2013). The phosphaturic mesenchymal tumor: why is definitive diagnosis and curative surgery often delayed?. Clin Orthop Relat Res.

[11] Mavrogenis AF, Sakellariou VI, Soultanis K, Mahera H, Korres DS, Papagelopoulos PJ (2010). A nonphosphaturic mesenchymal tumor mixed connective tissue variant of the sacrum. Orthopedics.

[12] Dey B, Gochhait D, Subramanian H, Ponnusamy M (2017). Oncogenic osteomalacia: an approach to diagnosis with a case report. J Clin Diagn Res.

[13] Zuo QY, Wang H, Li W (2017). Treatment and outcomes of tumor-induced osteomalacia associated with phosphaturic mesenchymal tumors: retrospective review of 12 patients. BMC Musculoskelet Disord.

